# Shelter and Housing Options, Supports, and Interventions for Older People Experiencing Homelessness

**DOI:** 10.1093/geroni/igaa057.2490

**Published:** 2020-12-16

**Authors:** Atiya Mahmood, Joe Humphries, Piper Moore, Victoria Burns, Sarah Canham

**Affiliations:** 1 Simon Fraser University, Vancouver, British Columbia, Canada; 2 College of Social Work, The University of Utah, Salt Lake City, Utah, United States; 3 University of Calgary, Calgary, Alberta, Canada; 4 University of Utah, Salt Lake City, Utah, United States

## Abstract

While older people experiencing homelessness (OPEH) can have life histories of homelessness or experience homelessness for the first time in later life, understandings of shelter/housing models that meet diverse needs of this population are limited. We conducted a scoping review of the international literature on shelter/housing models available to support OPEH. Through an iterative process of reading and rereading 24 sources (published 1999-2019), findings were organized into 5 categories of shelter/housing models that have been developed to support OPEH: 1) Permanent supportive housing (PSH), including PSH delivered through Housing First, 2) Transitional housing, 3) Shelter settings with medical supports, 4) Drop-in centers, and 5) Case management and outreach. Findings expand our understanding of how a continuum of shelter/housing options are needed to support distinct health and housing needs of diverse OPEH. Policy and practice implications related to integrating health and social care to support OPEH to age-in-the-right-place will be discussed. Part of a symposium sponsored by the Environmental Gerontology Interest Group.

